# Blood Levels of Co-inhibitory-Receptors: A Biomarker of Disease Prognosis in Multiple Sclerosis

**DOI:** 10.3389/fimmu.2019.00835

**Published:** 2019-04-30

**Authors:** Iris Lavon, Coral Heli, Livnat Brill, Hanna Charbit, Adi Vaknin-Dembinsky

**Affiliations:** ^1^Department of Neurology and Laboratory of Neuroimmunology, and the Agnes-Ginges Center for Neurogenetics, Hadassah-Hebrew University Medical Center, Jerusalem, Israel; ^2^Leslie and Michael Gaffin Center for Neuro-Oncology, Hadassah-Hebrew University Medical Center, Jerusalem, Israel

**Keywords:** co-inhibitory-receptors, multiple-sclerosis, precision medicine, circulating biomarkers, LAG-3, TIGIT, TIM-3

## Abstract

**Background:** The clinical course of multiple sclerosis ranges from benign with little disease progression and minimal disability, to severe disease requiring intensive medical treatment. There are no reliable circulating biomarkers for predicting disease outcome. Co-inhibitory receptors regulate the termination of effective immune responses to infections while limiting autoimmunity and/or immunopathology. Based on this, we studied the potential of circulating co-inhibitory receptor levels as predictive biomarkers of multiple sclerosis outcome.

**Methods:** Co-inhibitory receptor [TIGIT (T cell immunoreceptor with Ig and ITIM domains), TIM-3 (T-cell immunoglobulin and mucin domain–containing 3), LAG-3 (lymphocyte activation gene 3), PD-1 (programmed cell death 1), CTLA-4 (cytotoxic T-lymphocyte–associated protein 4)] expression levels in peripheral blood mononuclear cells (PBMCs) were measured using reverse transcription–PCR in 19 healthy controls and 57 patients with untreated multiple sclerosis. All patients were evaluated for disease outcome and paraclinical measures during the following 9–10 years [progression index, Expanded Disability Status Scale (EDSS) score, number of relapses, number of disease modifying therapies (DMTs), baseline brain magnetic resonance imaging T2 lesion volume, and oligoclonal bands (OCBs)].

**Results:** Patients had significantly lower TIGIT and LAG-3 levels than the controls (*P* < 0.02 and *P* < 0.04, respectively). TIM-3 levels were significantly lower in patients with high vs. low disability index and in patients with SPMS diagnosis compared to patients who remained in the relapsing stage of the disease at final visit (both, *P* < 0.02). LAG-3 levels were significantly higher in patients with low disability index vs. non-low disability index multiple sclerosis (*P* < 0.05). TIM-3 and LAG-3 expression levels correlated significantly with 1-year progression index (*r*^2^ = 0.076, *P* < 0.05; 0.087, *P* < 0.04, respectively) and EDSS score at final visit (*r*^2^ = 0.31, *P* < 0.04; 0.320.088, *P* < 0.04, respectively). Lower LAG-3 levels were associated with higher DMT switching (*r*^2^ = 0.67, *P* < 0.05). Compared to the paraclinical and clinical parameters alone, the combined data of the baseline co-inhibitory receptor expression levels and the paraclinical and clinical parameters were superior for predicting the patients that would progress to secondary progressive multiple sclerosis (SPMS).

**Interpretation:** This is an initial exploration of the utility of CTLA-4, PD-1, TIM-3, LAG-3, and TIGIT expression levels as prognostic indicators in untreated, recently diagnosed multiple sclerosis. Our results support the value of decreased PBMC expression levels of TIM-3 and LAG-3 at diagnosis as an unfavorable prognostic factor, which is to be confirmed in further studies.

## Introduction

Multiple sclerosis (MS) is a chronic autoimmune demyelinating disease of the central nervous system (CNS) directed against myelin proteins. The pathological process is characterized by neuroinflammation, causing extensive demyelination, gliosis, and axonal damage. Over two million people are affected by the disease worldwide, making MS the leading cause of neurological disability in young adults (1). MS is highly heterogeneous, as reflected by large variability in its clinical course, timing of relapses, and rate of disability progression. Although 15% of patients remain disability-free even decades after diagnosis and are defined as benign MS, over 50% of patients will need assistance in walking, and as many as 15% of patients with MS will have rapid progression, and reach a significant level of disability within a short period of time (1–3). The heterogeneity of the disease process complicates MS management, as the individual patient's disease course cannot be anticipated at diagnosis.

Today, there are more than 12 US Food-and-Drug-Administration–approved MS drugs with varied clinical response rates and safety profiles. Despite the advances in treatment options and acknowledging the disease heterogeneity, there is presently no objective parameter for predicting the disease course in MS. This is of great importance because there is a limited time window for effective intervention in MS patients with cumulative disability. Intervention during this period appears to be critical for achieving favorable long-term outcomes and preventing permanent neurological disability (4). Therefore, there is an unmet need for an objective circulating biomarker for predicting disease severity and prognosis and for aiding the physician in optimizing therapy for the individual patient.

Co-inhibitory-receptors such as CTLA-4 (cytotoxic T-lymphocyte–associated protein 4), LAG-3 (lymphocyte activation gene 3; or CD223), TIM-3 (T-cell immunoglobulin and mucin domain–containing 3), PD-1 (PDCD1; programmed cell death 1), and TIGIT (T-cell immunoreceptor with Ig and ITIM domains) are key factors in maintaining immune homeostasis and play a central role in regulating autoimmune diseases (5–7). These receptors regulate T-cell responses by inhibiting effector T-cell activation directly by promoting the suppressive function of regulatory T-cells (Tregs) and affecting antigen presentation. These cell surface molecules are expressed on activated immune cells (T-cells, B cells, natural killer [NK] cells, some myeloid cells) that regulate the inflammatory and autoimmune responses through a negative feedback mechanism. Malfunction of their crucial role or decreased receptor levels can lead to excessive immune activation and autoimmunity (5). While augmented effector T-cell activation plays a major role in MS pathogenesis, insufficient co-inhibitory signals might promote MS development and progression. In mice, CTLA-4 and PD-1 deficiency result in the development of spontaneous autoimmunity (8–10). Recent studies have also shown that multiple co-inhibitory-molecules, e.g., TIM-3, LAG-3, and TIGIT, predominantly regulate the effector T-cell responses within the tissue where their responses are executed (7). Based on the findings on the important role of the co-inhibitory-molecules in regulating autoimmunity and cancer immunity, it might be assumed that they also play a role in MS development and/or progression.

The present study was designed to test the possibility that these receptors can serve as markers of disease progression and disability, and in particular, whether their expression can aid the differentiation of patients with low vs. high disability indexes. In addition, we wanted to determine whether patients with relapsing remitting disease could be differentiated from patients who progress to secondary progressive MS (SPMS). We found that patients with MS had significantly lower TIGIT and LAG-3 levels than healthy controls (HC) and that TIM-3 and LAG-3 expression levels correlated significantly with MS outcome measures. Using these biomarkers will improve our ability to plan better treatment in individual patients with MS, leading to personalized medicine.

## Methods

### Subjects

We included MS patients who had been diagnosed with new-onset MS in 2007–2009. Their blood samples were collected and they were followed since diagnosis at the Hadassah MS Center and Department of Neurology at least twice annually. The Hadassah Medical Organization Ethics Committee approved this study. All patients and HCs provided written informed consent. 57 MS patients (41 women, 16 men; average age, 36.6 ± 10.8 years; baseline Expanded Disability Status Scale (EDSS) score, 1.4 ± 0.8) were eligible for analysis. The inclusion criteria were: untreated MS, diagnosis of not more than 2 years, and clinical data set accessibility [brain magnetic resonance imaging (MRI) at diagnosis, ≥6 visits with EDSS, including 10-year visit post-diagnosis]. A control group comprising 19 healthy individuals was used as a reference (11 women, 8 men; average age, 33.2 ± 10.8 years). Of 141 patients, 84 were excluded from the study because they did not fulfill the inclusion criteria and/or were missing longitudinal clinical data. EDSS score at final visit, number of relapses, and disease modifying drugs, oligoclonal bands (OCBs) were obtained from the patients' files. Of the patients, 72% had positive OCBs. Baseline MRI was performed after a mean of 4.7 ± 4.3 months following diagnosis (range, 0–12 months), and the patients' baseline brain MRI T2 mean lesion volume (T2LV) at the initial study visit was 20.6 cm^3^. Diagnoses were made in accordance to 2010 diagnostic criteria (2). Of the blood samples drawn and frozen in 2007–2009, patients were divided to 3 groups, according to the EDSS score 10 years after diagnosis, as indicated. Patients with EDSS score ≤ 1.5 were defined as low disability index (*n* = 22), patients with EDSS score ≥ 6 in <5 years from diagnosis (11) were defined high disability index (*n* = 17), and the rest of the cohort with EDSS score of 2–5.5 after 10 years was defined as medium disability index (*n* = 18). All patients in our cohort were diagnosed at first visit with relapsing remitting disease (RRMS). At the final visit, 37 patients still had RRMS, and 20 patients were diagnosed with SPMS.

### Collection of Peripheral Blood

Peripheral blood mononuclear cells (PBMCs) were isolated from freshly drawn heparinized blood by Ficoll-Paque (Amersham Pharmacia Biotech, Uppsala, Sweden) gradient centrifugation according to the manufacturer's protocol. The PBMCs were stored in TRI Reagent® (Sigma-Aldrich, Rehovot, Israel) at −80°C. RNA was extracted as described previously (12).

### Real-Time Reverse Transcription –PCR (RT-PCR)

Real-time RT PCR was performed on cDNA produced from 250 ng total RNA using SYBR Green as previously described (13). The fold changes (FCs) of the target mRNAs were normalized to *HPRT* (hypoxanthine phosphoribosyltransferase 1). Then, the FCs of each mRNA were calculated based on the ratio between the patient groups and HCs as indicated. The experiment was repeated three times in triplicate; the threshold cycle value (2^−ΔCT^) was used for statistical analysis and the results are presented as FC. We used the following primers:

*HPRT*: forward (F), 5′-TCCTCCTCAGACCGCTTTT-3′; reverse (R), 5′-CCTGGTTCATCATCGCTAATC-3′

LAG-3: F, 5′-TCACATTGGCAATCATCACAGTG-3′; R, 5′-CGTTCTTGTCCAGATACTGGAGT-3′

TIM-3: F, 5′-CTGCTGCTGCTACTACTTACAAGG-3′; R, 5′-AGACGGGCACGAGGTTCC-3′

TIGIT: F, 5′-GGAGGTCCTAGAAAGCTCAGTG-3′; R, 5′-CGATGACTGCTGTGCAGATGA-3′

CTLA-4: F, 5′-CATCCCTGTCTTCTGCAAAGCAA-3′; R, 5′-CAGTGGCTTTGCCTGGAGAT-3′

PD-1 (*PDCD1*): F, 5′-CGGCCAGGATGGTTCTTAGAC-3′; R, 5′-GAGAAGCTGCAGGTGAAGGT-3′.

### MRI Lesion Volume Analysis

Lesion volume was analyzed using the open source mrVista package (http://vistalab.standford.edu/software). For each patient, white matter lesions were manually segmented on fluid-attenuated inversion recovery (FLAIR)-weighted images and their volume was extracted (14).

### Statistical, Heat Map, and Principle Component Analysis (PCA)

The data were analyzed using Student's *t*-test, one-way analysis of variance, and Spearman's correlation. *P* < 0.05 was considered statistically significant. All data are presented as the mean ± SE. To reveal potential unsupervised clustering, we performed principle component analysis (PCA) and heat map analysis using ClustVis software as demonstrated by Metsalu and Vilo (15).

Specificity and sensitivity were calculated using MedCalc (2019 MedCalc Software bvba). The risk of reaching an EDSS score of 3 or 6 (as indicated) was estimated using Kaplan–Meier curves. The survival curves were constructed using R version 2.43-3 (available from http://astatsa.com/LogRankTest/), R package “survival” (16).

## Results

### Co-inhibitory-Receptor Levels in HCs and MS Patients

**Figure 1:**
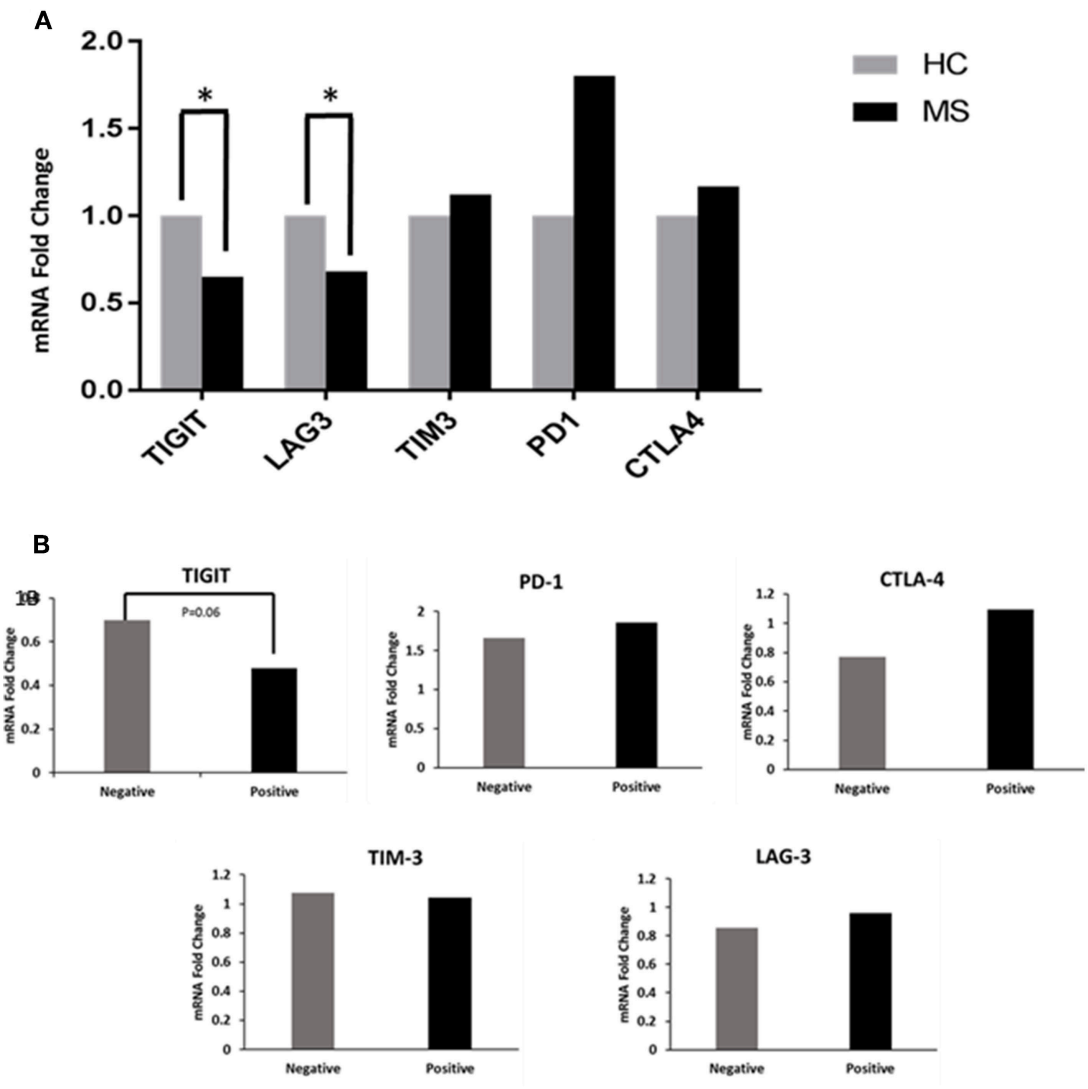
Co-inhibitory receptor levels in HCs and patients. **(A)** RT-PCR determined the FC of TIGIT, LAG-3, TIM-3, PD-1, and CTLA-4 mRNA in PBMCs of patients (n = 57) compared to HCs (*n* = 19). TIGIT and LAG-3 expression levels were significantly decreased in patients (^*^P < 0.02 and ^*^P < 0.04). **(B)** Decreased TIGIT expression in OCB-positive vs. OCB-negative patients (*P* = 0.06).

MS patients had significantly decreased TIGIT and LAG-3 expression levels (0.68 and 0.65 FC) as compared to the HCs (1 FC) (*P* < 0.02 and *P* < 0.04, Figure 1A). The TIM-3, PD-1, and CTLA-4 levels between the MS patients and HCs were not statistically significantly different. When the MS patients were grouped according to OCB status, there was a trend toward decreased TIGIT expression in OCB-positive patients (0.48 FC) compared to OCB-negative patients (0.70 FC) (*P* = 0.06, Figure 1B).

### LAG-3 and TIM-3 Levels Predicted Disease Course

**Figure 2:**
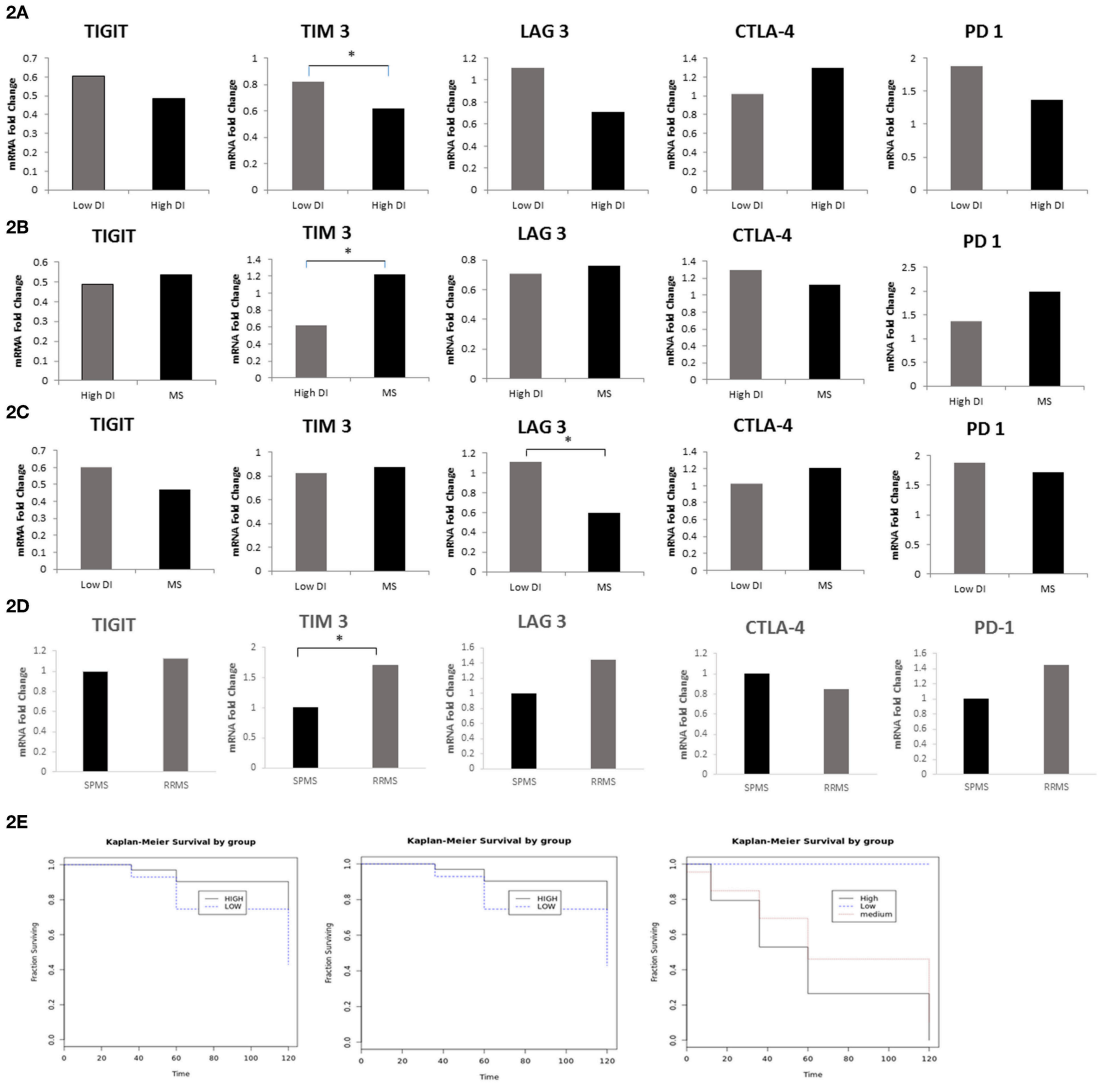
LAG-3 and TIM-3 Levels Predict MS Disease Course. FC of TIGIT, PD-1, CTLA-4, LAG-3, and TIM-3 mRNA in PBMCs in **(A)** MS with the worst outcome (high disability index patients as compared to the patients group with low disability index (^*^P < 0.02); **(B)** high disability index patients compared to all other MS (^*^P < 0.02); **(C)** low disability index patients compared to all other MS (^*^P < 0.05); **(D)** patients that still had RRMS after 10 years (*n* = 37) as compared to patients that progressed to SPMS (*n* = 20) (^*^P < 0.02); **(E)** Kaplan–Meier curves showing time to reach a specific EDSS score. Patients with low expression levels of TIM-3 (i) and LAG-3 (ii) (dashed line) were compared with patients with high TIM-3 and LAG-3 levels (bolded line) (*P* < 0.005); (iii) Kaplan–Meier curves of the three groups in the study: patients with low disability index (dashed line), patients with medium disability index (dotted line), and patients with high disability index (bolded line).

Subsequently, we investigated whether co-inhibitory-receptor expression levels in MS patient PBMCs measured around the time of diagnosis could predict the disease outcome. We first compared the relative expression (FC) of TIGIT, PD-1, *CTLA4*, LAG-3, and TIM-3 in the worst MS outcome (patients with high disability index, EDSS score of 6 in ≤5 years after diagnosis) to that of patients with mild MS (low disability index, EDSS score ≤1.5 at 10 years after diagnosis). Patients with high disability index had significantly lower TIM-3 levels and a trend for lower LAG-3 levels compared to patients with low disability (MS with high vs. low disability index: TIM-3: 0.61 FC vs. 0.82 FC, *P* < 0.02; LAG-3: 0.7 FC vs. 1.1 FC, *P* < 0.07) (Figure 2A). Furthermore, patients with high disability index had significantly lower levels of TIM-3 compared to all other patients: 0.61 FC vs. 1.15 FC, *P* < 0.02, Figure 2B). Moreover, LAG-3 levels were significantly higher in patients with low disability index vs. all other MS forms: 1.1 FC vs. 0.6 FC, *P* < 0.05, Figure 2C).

All patients in our cohort were diagnosed at the first study visit with RRMS. We investigated whether the expression levels of co-inhibitory receptors measured around the time of diagnosis could differentiate between patients who, after 10 years, remained in the relapsing remitting stage (*n* = 37) and those who progressed to the secondary progressive stage (*n* = 20). We found that patients with SPMS at the final visit had significantly lower levels of TIM-3 and a trend toward lower LAG-3 levels compared to patients who remained in the relapsing stage (SPMS vs. RRMS: 1 FC vs. 1.7 FC, *P* < 0.02, 1 FC vs. 1.45 FC, *P* < 0.06 TIM-3 and LAG-3, respectively, Figure 2D). The Kaplan–Meier method was used to study the distributions of time to reach an EDSS score of 6, stratified by high (higher than average) expression levels of TIM-3 and LAG-3 as compared to low expression levels. Patients with lower, as opposed to higher, TIM-3 or LAG-3 expression reached an EDSS score of 6 significantly earlier (both, *P* < 0.005) (Figure 2E).

### Co-inhibitory-Receptors and Clinical and Paraclinical Variables

**Figure 3:**
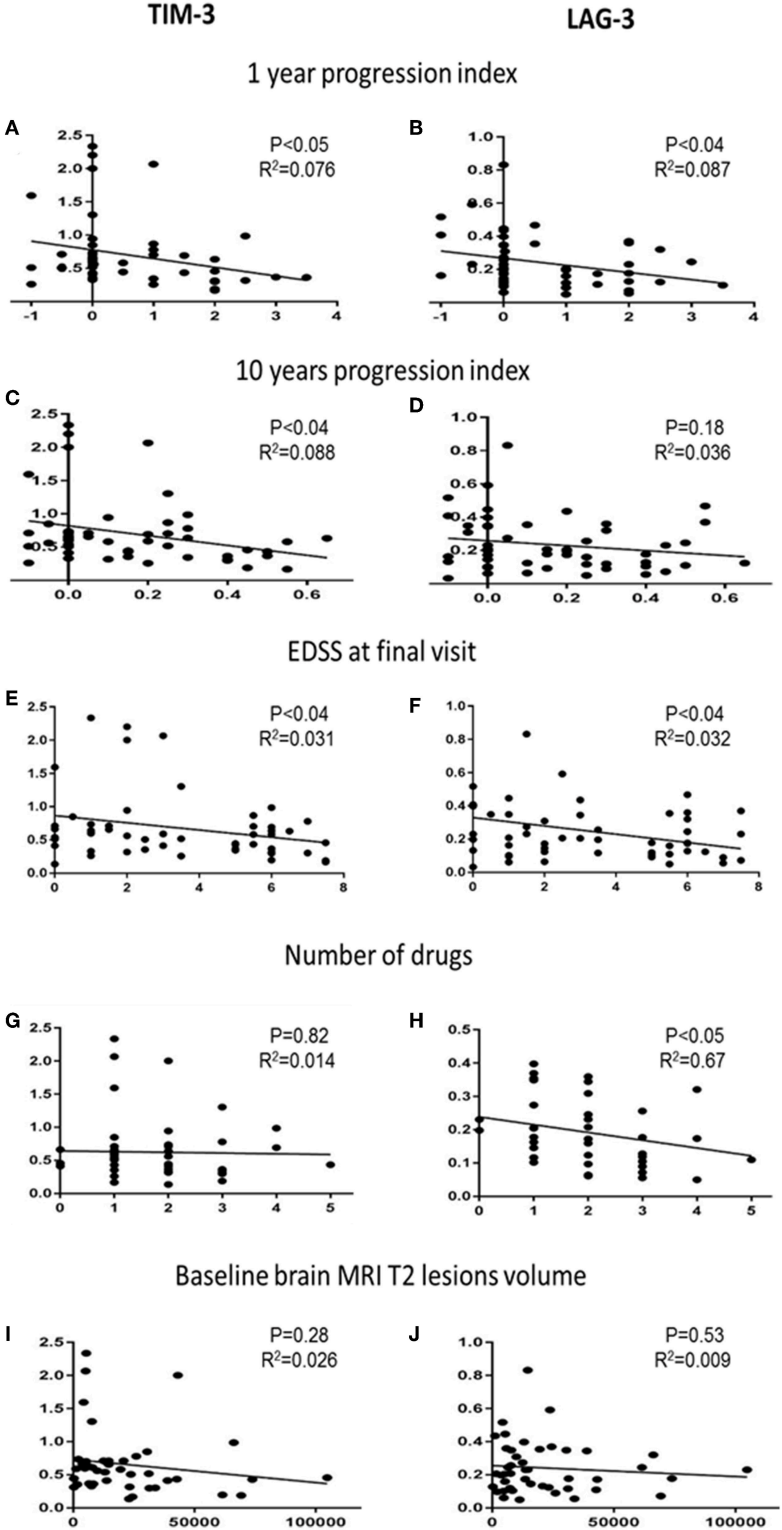
Co-inhibitory receptors and clinical and paraclinical variables. Correlation analysis of TIM-3 and LAG-3 expression levels with 1-year progression indexes **(A,B)**, 10-year progression indexes **(C,D)**, EDSS score at final visit **(E,F)**, number of DMTs used by each patient **(G,H)**, and T2LV at baseline **(I,J)**.

Next, we investigated whether the co-inhibitory-receptor expression levels were associated with clinical disease progression measurements [1- and 10-year progression index, EDSS score at final visit, number of disease-modifying therapy (DMT) switches during the 10-year follow-up, number of relapses, baseline brain MRI T2 lesion volume (T2LV)] (Figure 3 and Supplementary Figure 1).

The 1-year progression indexes correlated significantly with TIM-3 (*r*^2^ = 0.076, *P* < 0.05) and LAG-3 (*r*^2^ = 0.087, *P* < 0.04) expression levels; the 10-year progression indexes correlated significantly with TIM-3 expression levels (*r*^2^ = 0.088, *P* < 0.04) (Figures 3A–C). TIM-3 and LAG-3 expression levels and EDSS score at final visit were also significantly correlated (*r*^2^ = 0.31, 0.32; both, *P* < 0.04, Figures 3E,F).

The cohort included newly diagnosed patients who were followed for 8–10 years. Most patients started therapy shortly after the baseline blood sample had been obtained, and several switched therapy during the follow-up. Each patient's number of DMTs correlated to the expression levels of the co-inhibitory-receptors. We found a significant negative correlation between LAG-3 expression and the number of DMTs. Lower LAG-3 levels were associated with higher DMT switching (Figure 3H, *r*^2^ = 0.67, *P* < 0.05).

Among the available MS diagnosis parameters with potential prognostic value, brain T2LV is of particular importance. We therefore correlated the baseline T2LV with the co-inhibitory-receptor expression levels. There was no association between baseline T2LV and the individual co-inhibitory-receptor expression level (Figures 3I,J). In addition, no association was found between the number of relapses during the follow-up and the co-inhibitory-receptor expression levels in the patients (Supplementary Figure 2). We also did not detect any association between co-inhibitory-receptor expression levels and age or sex.

### LAG-3 and TIM-3 Expression Levels in the Peripheral Blood at Diagnosis Can Predict MS Patients' Outcome

**Figure 4:**
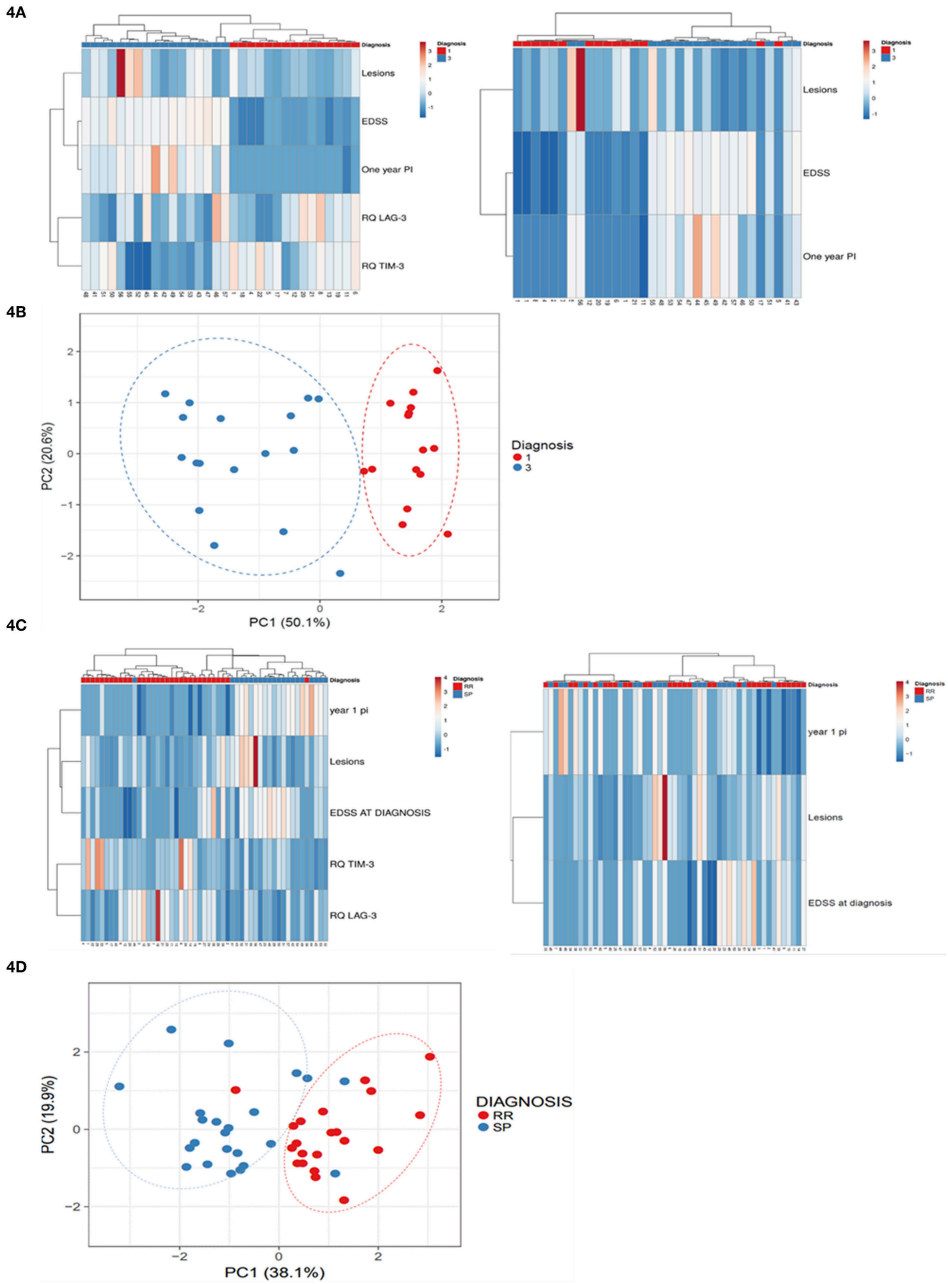
Baseline LAG-3 and TIM-3 Expression Levels combined with paraclinical and clinical data predicted high disability index patients vs. low disability index patients and conversion to SPMS. Unsupervised heat map analysis **(A,C)** and principle component analysis (PCA) **(B,D)** using ClustVis software on the raw data of 1-year progression index (1 year PI), baseline MRI lesion volume (Lesions), EDSS at diagnosis (EDSS) combined with LAG-3 and TIM-3 expression levels. **(A,C)** Unsupervised heat map analysis. Rows are centered; unit variance scaling is applied to rows. Both rows and columns are clustered using correlation distance and average linkage. Each row represent clinical or gene expression parameters (as indicated). Each column represents sample of one patient. Decreased gene expression is displayed in blue, **(B,D)** PCA: SVD (singular value decomposition) with imputation was used to calculate the principal components. X-axis, principal component 1; Y-axis, principal component 2, which explain 50.1 and 20.6% of the total variance *N* = 32 **(B)**, and 19.9 and 38.1% of the total variance **(D)**, respectively. Ellipses represent the 95% confidence interval. *N* = 53 data points. Red, low disability index patients; blue, high disability index patients.

To reveal potential unsupervised clustering, we performed heat map analysis and PCA using ClustVis software (15) on the raw data available early in the disease course, i.e., 1-year progression index, EDSS at diagnosis and baseline MRI lesion volume, combined with LAG-3 and TIM-3 expression levels (Figure 4). We first analyzed data obtained from the patients with high and low disability index. The unsupervised heat map demonstrated that the combined data of the LAG-3 and TIM-3 expression levels and the paraclinical and clinical parameters clearly separated the patients into two distinct groups which reflect the disease disability index (Figure 4A; left panel), PCA, another unsupervised analysis method, validated this result (Figure 4B). More importantly, unsupervised analysis of all patients revealed that the combined data of the LAG-3 and TIM-3 expression levels and the paraclinical and clinical parameters clearly separated the patients who still had RRMS from those who progressed to SPMS (Figure 4C, right panel, Figure 4D).

The combined data of the baseline co-inhibitory receptor expression levels and the paraclinical and clinical parameters were superior for predicting the patients that would progress to SPMS. The combined parameters had 95.24% sensitivity and 96.88% specificity compared to the paraclinical and clinical parameters alone, which had 66.67% sensitivity and 40% specificity. The addition of the baseline expression levels of the co-inhibitory receptors to the paraclinical and clinical parameters also increased the ability to predict the patients that would have severe vs. mild disease outcome. Sensitivity and specificity were both 100% compared to the 88% sensitivity and 89% specificity when predictive ability was calculated by the paraclinical and clinical parameters alone.

## Discussion

Our main finding is that LAG-3 and TIM-3 expression levels in the PBMCs of MS patients can aid to predict disease outcome. Lower LAG-3 and TIM-3 expression were associated with the worst outcome and increased likelihood to progress to SPMS, while higher LAG-3 and TIM-3 expression correlated significantly with benign prognosis, persisting RRMS, and lower EDSS score at 10 years after blood sampling. Analysis of the paraclinical and clinical parameters baseline brain MRI T2LV, EDSS at diagnosis, and 1-year progression index combined with LAG-3 and TIM-3 expression levels resulted in superior ability to identify patients who would progress to SPMS and patients with high disability index as compared to the paraclinical and clinical parameters alone. Furthermore, the PBMCs of the patients had significantly lower TIGIT and LAG-3 expression compared to that of the HCs. Patients with OCB-positive CSF had lower TIGIT levels compared to patients with OCB-negative CSF. TIGIT is an inhibitory immunoreceptor expressed by T and NK cells. There are no data on the role of TIGIT in MS, but Tigit-overexpressing transgenic mice or mice treated with a TIGIT agonistic antibody had reduced experimental autoimmune encephalomyelitis (EAE) induction, whereas TIGIT-deficient mice were more susceptible to EAE (17). There are few data on the role of LAG-3 in MS and EAE. LAG-3 is structurally similar to the CD4 molecule that binds major histocompatibility complex class II molecules and inhibits activated T-cells. LAG-3 polymorphism has been associated with MS (13). Kadowaki et al. showed that the gut epithelium of MOG (myelin oligodendrocyte glycoprotein) (35–55)-specific T-cell receptor transgenic mice contained induced intraepithelial lymphocytes that inhibit EAE. These cells infiltrated the CNS and inhibited inflammation mainly by marked LAG-3 upregulation (18). Another study revealed that LAG-3 acts synergistically with PD-1 to prevent autoimmunity in mice (8). These findings support our results, indicating that lower LAG-3 expression correlates with more severe MS outcome.

TIM-3 was first identified as a T-cell surface molecule expressed selectively in interferon (IFN)-γ-producing T-cells (19). It is also expressed in innate immune cells (dendritic cells, NK cells, monocytes) and Tregs (7). TIM-3 blockade worsens EAE symptoms (19). Several TIM-3 gene single-nucleotide polymorphisms, as well as abnormalities in TIM-3 regulation, have been identified in MS (20, 21). Koguchi et al. demonstrated dysregulated TIM-3 expression in MS CSF clones (20). Interestingly, preliminary genome-wide analysis of mRNA expression data from the peripheral blood of patients with MS showed that TIM-3 is significantly induced in responders to IFN-β therapy, whereas non-responders show lower or no TIM-3 induction (22). The finding of increased TIM-3 levels in patients with better response to therapy supports our assumption that TIM-3 levels may determine disease outcome.

The co-inhibitory receptors that either differ significantly in patients with MS as compared to the HCs (TIGIT, LAG-3) or that predict MS outcome (LAG-3 and TIM-3) are associated with the immunoregulatory cytokine interleukin IL-10 (23–25). It is well-described that IL-10 is decreased in MS (22, 26). The decreased co-inhibitory-receptors found in the present study might therefore cause ineffective Treg-mediated suppression via reduced IL-10 production. This could be one reason for increased effector T-cell function and worse disease outcome.

The most studied co-inhibitory-receptors in MS and EAE are CTLA-4 and PD-1. Blocking CTLA-4 in EAE mice resulted in a significantly severe clinical course and more inflammatory and demyelinating lesions in the CNS. It also enhanced epitope spreading and increased cytokine production (22, 26). A recent study has suggested that CTLA-4 may also intrinsically limit Treg activation and expansion and that deletion of CTLA-4 in Treg during adulthood protects against EAE (22). The contradictory role of CTLA-4 in the T effector and regulatory cells may explain the lack of correlation between its expression levels and MS severity.

Polymorphisms in the PD-1 gene locus appear to be associated with disease progression in MS (27), and mice deficient in PD-1 and PD-L1 (PD-1 ligand) were more susceptible to induced EAE than a wild-type control (28). However, we did not observe correlation between PD-1 expression levels and MS severity.

The main characteristic of T-cell exhaustion that contributes to tumor development is the enhanced expression of immune checkpoints. Following this remarkable discovery, there has been increased usage of co-inhibitory-receptor blockage therapy for treating cancer. As a result of the removal of self-tolerance by these treatments, there is increased autoimmunity risk, termed immune-related adverse events (29). Neurologic immune-related events are rare and include inflammatory myopathy, myelitis, Guillain-Barré syndrome, encephalitis, and myasthenia gravis (30). Several case reports have described MS exacerbations, and one case of MS development (in a patient with a preexisting subclinical disease known as radiologically isolated syndrome) following anti–CTLA-4 therapy (31). Although we did not observe decreased CTLA-4 in the patients, the above clinical observations support the hypothesis that co-inhibitory-mechanisms may be involved in MS development and/or outcome (32).

The current MS treatment recommendations are that DMTs should be initiated at an early disease stage because they are likely to influence future disease outcome [reviewed in Freedman et al. (33)]. There are currently more than a dozen DMTs available for MS, with different modes of action, adverse effects profiles, and response rates, making differential therapy protocol possible for patients with mild disease as opposed to those with probable worse disease outcome. It is believed that a biomarker that enables the clinician to identify which patients will need a more effective DMT is crucial for providing more individualized treatment.

Natural history studies have revealed that both male gender and OCB-positive CSF are associated with greater accumulation of disability, whereas patients in whom the first relapse is optic neuritis will have lower risk of accumulation of disability compared with patients displaying other types of relapse (34–38). Most clinicians use brain MRI to predict future accumulation of disability. In a recent study, Popescu and colleagues found that brain atrophy and baseline T2LV could predict MS long-term disability (39, 40). There is also a known correlation between brain volume loss and T2LV (41). Recent works have revealed the importance of baseline MRI for predicting disease evolution. Tintore et al., Kuhle et al., and Comabella et al. found that the number of lesions on brain MRI at baseline has the highest impact as a disease prognostic factor (42–44). Nevertheless, MRI findings and disease severity are not correlated in many patients with MS. This is known as the clinico-radiological paradox or dissociation (45). Here, we found that analysis of baseline brain MRI T2LV at baseline, EDSS at diagnosis and 1-year progression index combined with LAG-3 and TIM-3 expression levels resulted in superior ability to identify patients with poor outcome as compared to baseline brain MRI T2LV, EDSS at diagnosis and 1-year progression index alone. However, no direct correlation between co-inhibitory-receptor expression levels and brain MRI T2LV was observed.

Recently, several circulating biomarkers have been identified in MS. The most widely investigated are neurofilament light (NF-L), which are unique to neuronal cells. CSF as well as serum levels of NF-L were associated with disease measurements such as EDSS, relapse rate, as well as brain atrophy and disability progression in early MS (46–48).

Hypothetically, combining all known biomarkers might improve our ability to better foretell disease activity and would facilitate better personalized treatment for MS. Nevertheless, implementing the use of MS biomarkers requires vigorous clinical and multicenter validation. Based on our findings, we suggest that decreased PBMC expression levels of TIM-3 and LAG-3 at MS diagnosis may represent an unfavorable prognostic factor.

## Ethics Statement

The study approved by Hadassah Medical Organization Ethics Committee.

## Author Contributions

IL contributed to the analysis of the results and to the writing of the manuscript. CH performed the experiments. LB contributed to the design and implementation of the research. HC collected the samples. AV-D contributed to the design and implementation of the research, to the analysis of the results and to the writing of the manuscript. All authors discussed the results and commented on the manuscript.

### Conflict of Interest Statement

The authors declare that the research was conducted in the absence of any commercial or financial relationships that could be construed as a potential conflict of interest.

## Supplementary Material

Supplementary Figure 1 Additional Co-Inhibitory Receptors and Clinical and Paraclinical Variables. Correlation analysis of TIGIT, PD-1, and CTLA-4 expression levels with 1-year progression indexes **(A–C)**, 10-year progression indexes **(D–F)**, EDSS score at final visit **(G–I)**, number of DMTs used by each patient **(J–L)**, and T2LV at baseline **(M–O)**.

Supplementary Figure 2 Association between the number of relapses during the follow-up and the co-inhibitory-receptor expression levels in the patients.
