# Supplementary figures and images for: Blood Levels of Co-inhibitory-Receptors: A Biomarker of Disease Prognosis in Multiple Sclerosis
Source: Front Immunol. 2019 Apr 30;10:835. doi: 10.3389/fimmu.2019.00835 (PMC6524709; doi:10.3389/fimmu.2019.00835)

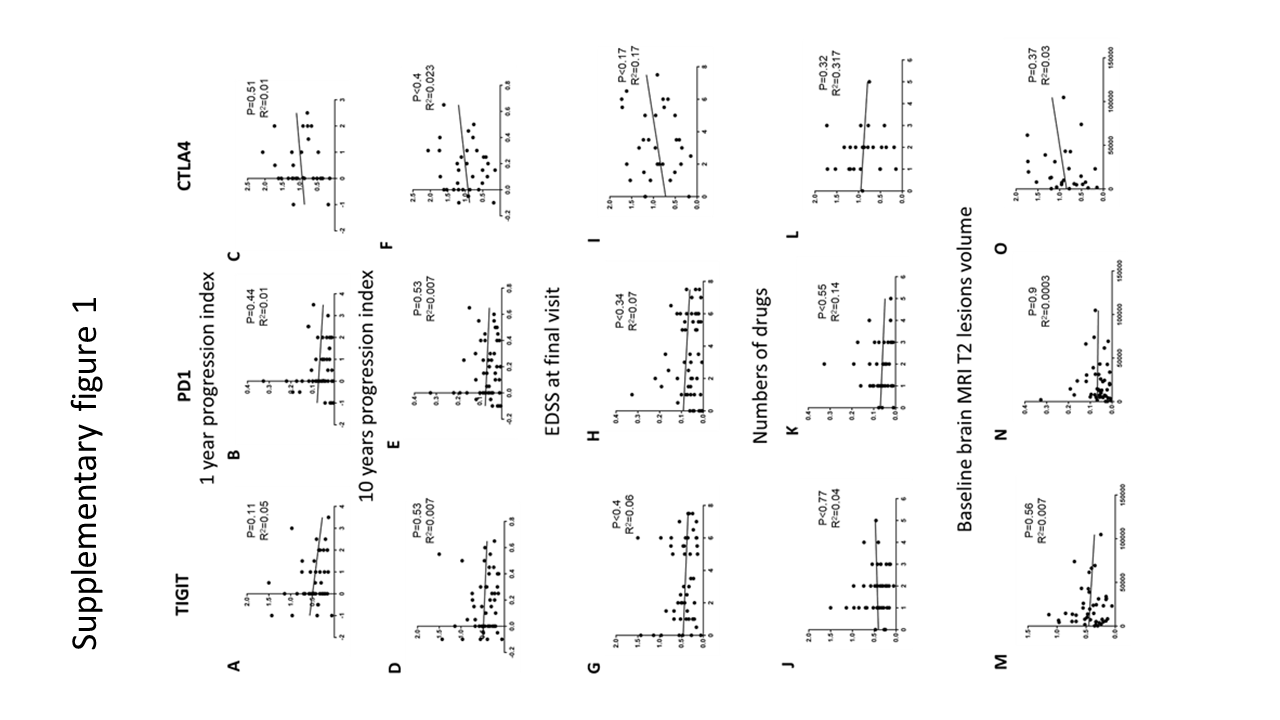

Supplement: Supplementary Figure 1 — Additional Co-Inhibitory Receptors and Clinical and Paraclinical Variables. Correlation analysis of TIGIT, PD-1, and CTLA-4 expression levels with 1-year progression indexes (A–C), 10-year progression indexes (D–F), EDSS score at final visit (G–I), number of DMTs used by each patient (J–L), and T2LV at baseline (M–O). [file Image_1.tif]

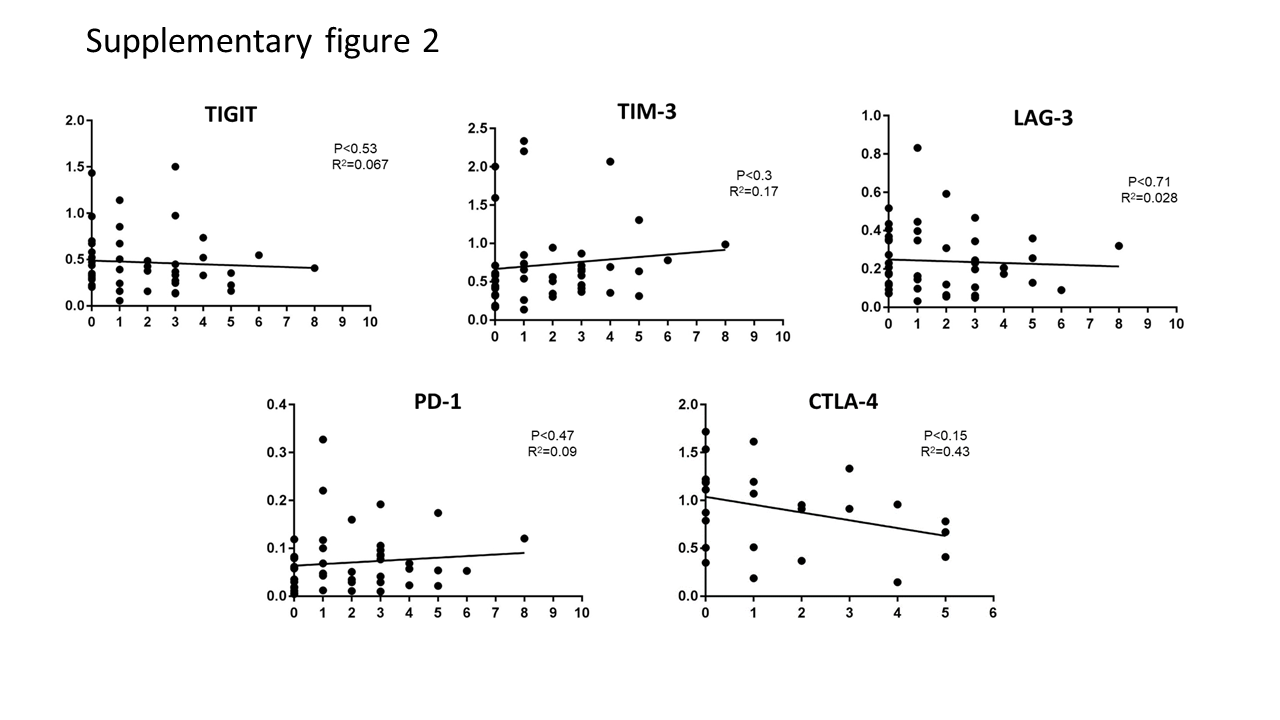

Supplement: Supplementary Figure 2 — Association between the number of relapses during the follow-up and the co-inhibitory-receptor expression levels in the patients. [file Image_2.tif]
